# Towards a Multispectral Imaging System for Spatial Mapping of Chemical Composition in Fresh-Cut Pineapple (*Ananas comosus*)

**DOI:** 10.3390/foods12173243

**Published:** 2023-08-28

**Authors:** Kaveh Mollazade, Norhashila Hashim, Manuela Zude-Sasse

**Affiliations:** 1 Department of Biosystems Engineering, Faculty of Agriculture, University of Kurdistan, Sanandaj 6617715175, Iran; k.mollazade@uok.ac.ir; 2 Department of Horticultural Engineering, Leibniz Institute for Agricultural Engineering and Bioeconomy (ATB), 14469 Potsdam, Germany; 3 Department of Biological and Agricultural Engineering, Faculty of Engineering, Universiti Putra Malaysia, Serdang 43400, Selangor, Malaysia; norhashila@upm.edu.my; 4 SMART Farming Technology Research Centre, Faculty of Engineering, Universiti Putra Malaysia, Serdang 43400, Selangor, Malaysia

**Keywords:** dimensionality reduction, hypercube, quality evaluation, wavelength selection

## Abstract

With increasing public demand for ready-to-eat fresh-cut fruit, the postharvest industry requires the development and adaptation of monitoring technologies to provide customers with a product of consistent quality. The fresh-cut trade of pineapples (*Ananas comosus*) is on the rise, favored by the sensory quality of the product and mechanization of the cutting process. In this paper, a multispectral imaging-based approach is introduced to provide distribution maps of moisture content, soluble solids content, and carotenoids content in fresh-cut pineapple. A dataset containing hyperspectral images (380–1690 nm) and reference measurements in 10 regions of interest of 60 fruit (*n* = 600) was prepared. Ranking and uncorrelatedness (based on ReliefF algorithm) and subset selection (based on CfsSubset algorithm) approaches were applied to find the most informative wavelengths in which bandpass optical filters or light sources are commercially available. The correlation coefficient and error metrics obtained by cross-validated multilayer perceptron neural network models indicated that the superior selected wavelengths (495, 500, 505, 1215, 1240, and 1425 nm) resulted in prediction of moisture content with R = 0.56, MAPE = 1.92%, soluble solids content with R = 0.52, MAPE = 14.72%, and carotenoids content with R = 0.63, MAPE = 43.99%. Prediction of chemical composition in each pixel of the multispectral images using the calibration models yielded spatially distributed quantification of the fruit slice, spatially varying according to the maturation of single fruitlets in the whole pineapple. Calibration models provided reliable responses spatially throughout the surface of fresh-cut pineapple slices with a constant error. According to the approach to use commercially relevant wavelengths, calibration models could be applied in classifying fruit segments in the mechanized preparation of fresh-cut produce.

## 1. Introduction

Pineapple, *Ananas comosus* L., is the third most important tropical fruit, in terms of international trade, after banana and citrus. Pineapple is a good source of vitamin C, provitamin A and vitamin B, contains nutritionally valuable minerals such as calcium, magnesium, phosphorus, and iron, and, most important for fresh-cut products the circular use of by-products is under development in much of the research undertaken at present [1,2]. Therefore, pineapple is considered one of the most critical commercial fruits in the world, with demand seeing a static rise year after year throughout the past decade, a phenomenon practically unique in the fruit sector [3]. Due to its large size, firm texture, and time-saving process of mechanized peeling, pineapple is a fruit suitable for minimal processing in the form of fresh-cut. Fresh-cut fruit are products that are partially processed (including peeling, slicing, dicing, or chopping) and are either ready to consume or packaged.

Fresh-cut pineapple may be offered to the market in either canned form or in regular packaging. The factors that affect the quality of fresh-cut pineapple include the cutting method, sanitation in preparation, the packaging technique, cold storage chain, and fruit chemical composition. Among these, the latter, because of affecting the appearance and aroma, is of great importance for consumers [4,5]. Pineapple develops a low respiration rate due to the non-climacteric traits and good storability of the fruit. In recent years, new packaging methods based on modified atmosphere conditions have been developed in order to further increase the shelf life of fresh-cut pineapple and particularly to maintain visual quality and to reduce microbial growth [6,7]. The success of these methods is dependent on the initial quality and level of chemical composition of pineapple [8]. Achieving this goal of prolonged shelf life requires technologies that can non-invasively determine the visual quality and quality-related chemical composition of fresh-cut pineapple before packaging as fresh-cut. In this way, it is possible to remove fruit or fruit parts of lower quality or aim at conforming pieces in one package before packaging. The morphology of the fruit is challenging, which consists of many individual fruitlets developing from flowers showing various flowing time. The variation in the developmental stage of fruitlets may lead to gradients of chemical composition within the entire fruit. The intra-fruit variation of chemical properties results in varying raw materials for the fresh-cut product [9].

Non-destructive spectroscopy was applied earlier in the pineapple to analyze fruit quality [10] and physiological disorders [11] based on ‘point’ spectroscopy. However, the spatial variability of pineapple is requested to consider the spatial variability within the fruit. Therefore, an imaging sensor system would be necessary. In the past two decades, hyperspectral imaging has been widely used for the quality assessment of fruit, vegetables, and other food materials due to its ability to simultaneously acquire spatial and spectral information and generate maps of chemical composition [12,13]. Various studies have reported the performance of predictive/classification models developed with data acquired from hyperspectral imaging. However, due to the time-consuming image acquisition, big size of acquired data (hypercubes), and equipment cost, hyperspectral imaging cannot be used for real-time applications, which require high speed in production lines. Instead, hypercubes can be analyzed to find wavelengths showing the highest correlation between spectral data and quality indices of interest. This led to the development of multispectral imaging systems, which compared to hyperspectral imaging systems, are not only cheaper but also suitable for online applications [14].

Since a multispectral image consists of several image channels, each taken from a specific wavelength, it can be acquired using filter-based or light-based methods. In the filter-based approach, the scene is illuminated by a broad-band light source in the desired wavelength range. Then, successive images of the scene are captured by a camera equipped with a wheel containing bandpass optical filters of the selected wavelengths. In the light-based approach, the scene is illuminated successively by different light sources (such as LED or divergent laser light) emitting light only at the selected wavelengths and then multispectral images are created by combining the acquired monochromatic images [15]. Although numerous studies have been conducted to find the optimal wavelengths for predicting various quality indices of fruit and vegetables [16,17], what remained incomplete in most of the research is the disregard for the availability/accessibility of the filters or light sources commercially in the chosen wavelengths and also, sometimes, the large number of chosen wavelengths. However, the economic success of an application of the technology depends on the availability and costs of the system components, which means filters or light sources need to be commercially available in the selected wavelengths, and secondly, the number of selected wavelengths is as low as possible. Furthermore, the visible and shortwave near infrared wavelength range is preferred, since the receivers are available at a lower cost. Therefore, it is necessary to develop methods for selecting the optimal wavelengths that consider both the economic aspect and the feasibility of developing a multispectral system based on the selected wavelengths.

Consequently, this research aimed to introduce a practical approach to create a multispectral system for generating distribution maps of chemical composition in fresh-cut pineapple. The aim of the study was approached through the following objectives: (i) registering the hyperspectral data with RGB images to find the spatially distributed measuring points of chemical analysis, (ii) selecting wavelengths where not only bandpass optical filters or light sources (LED or laser) are commercially available, but also showing more correlation between spectral data and changes in the chemical composition of the pineapple fruit, and (iii) developing regression models based on the selected wavelengths and generating distribution maps of chemical composition in fresh-cut pineapple.

## 2. Materials and Methods

### 2.1. Data Acquisition

In order to capture variation in the chemical composition of the fruit, 60 fresh pineapples of different varieties, produced in different geographical origins in Costa Rica (10 fruit from each origin), were bought from a fruit import company in Potsdam, Germany. Immediately after transferring pineapples into the laboratory, some physical properties were measured, including mass (g), height (cm), and arithmetic mean diameter (cm). In the middle part of each fruit, a 30-mm-thick slice was made along the stem axis. A sharp knife was used for cutting, and the utmost care was taken to avoid damaging the flesh of the fruit in the sliced area. Immediately after cutting the samples, hyperspectral images were acquired. Then, ten cylindrical samples were mechanically extracted using a cork borer (diameter = 18 mm) from different positions of each slice following a similar pattern [18]. Afterwards, an RGB image of the fruit slice, in which the locations of extracted cylindrical samples were identified, was taken. Subsequently, each cylindrical sample (*n* = 600) was divided into two sections: one section was used to measure the moisture content (MC, in %) by oven-drying (85 °C) until the weight remained stable, usually after 48 h [19]. The water content was calculated by the mass difference as a percentage of mass before drying. The other section was subjected to refractometric (DR 301-95, A. Krüss Optronic, Germany) readings of soluble solids content (SSC, in %), titratable acidity (TA) analyzed by titration at pH of 8.2 (citric acid, in g/100 mL), and carotenoid content (CC, in mg/100g DM). To measure total carotenoids content, first, the flesh tissue of fruit underwent homogenization using an UltraTurax T18 homogenizer from IKA Works, USA, with the continuous addition of 80% acetone from Roth, Germany, and a small amount of calcium carbonate. This process effectively separated the organically dissolved pigments from the remaining tissue, which were then collected using a G3 filter and combined to reach a 100 mL volume of acetone-pigment extract. To transfer the chromophoric compounds from the polar phase in acetone to the hexane phase in diethyl ether, a small amount of water and 25 mL of diethyl ether from Merck, Darmstadt, were added. The spectral absorption of this phase was recorded in cuvettes with 10 mm pathlength in transmittance geometry within the wavelength range of 350–850 nm with 0.5 nm resolution (Lambda 950, Perkin Elmer, Waltham, MA, USA). From the spectra obtained, carotenoids’ contents were calculated using the iterative multiple linear regression, iMLR, and related to the dry mass of the fruit’s cylindrical sample [20].

### 2.2. Hyperspectral Imaging

A hyperspectral imaging system in the visible-near infrared (VIS-NIR) wavelength range was used to acquire hypercubes in reflectance mode. This system consists of two spectrographs and two cameras: one with a working range of 380 to 1000 nm (Spectrograph: ImSpector V10E, spectral resolution: 2.8 nm, Specim Spectral Imaging Ltd., Oulu, Finland; Camera: Pixelfly qe b/w, 696 × 512 pixels, PCO AG, Kehlheim, Germany) and the other with a working range of 860 to 1690 nm (Spectrograph: ImSpector N17E, spectral resolution: 5 nm, Specim Spectral Imaging Ltd., Oulu, Finland; Camera: Xenics XEVA-USB 2.0, 320 × 256 pixels, Xenics, Leuven, Belgium). The lighting unit was composed of a stable light source (Model 3900 (Smart-Lite), 150 W halogen lamp, Spectral Output: 400–2200 nm, Illumination Technologies Inc., Onondaga County, NY, USA), an optical fiber, and a linear light deflector. Hypercubes were acquired at a distance of 60 cm from the surface of fruit slices by the push-broom method.

In this system, instead of moving the samples against the spectrograph, a two-mirror mechanism was employed as described earlier [21]. This mechanism provides the possibility of scanning the scene only by rotating the mirrors without need of movement of sample or spectrograph. One of the mirrors was fixed, in which the image of the scene was reflected. The second mirror, which was placed at an angle to the fixed mirror, was movable and its rotation was provided by a stepper motor. By rotating the movable mirror and changing its angle with respect to the fixed mirror, the scene in front of the spectrograph slit is changed and thus a linear scan of the scene is provided. Line-by-line scanning continued until the spectral signature of each fruit slice was acquired. In order to increase the signal-to-noise ratio, hyperspectral imaging was performed in a dark room, and the exposure time was set between 20 to 30 milliseconds. The control of hyperspectral imaging system was performed by using an in-house software (HyBis 1.0 datalog, ATB, Potsdam, Germany), which was developed in LabVIEW (ver. 8.6, National Instruments, Austin, TX, USA).

### 2.3. Image Registration

**Figure 1:**
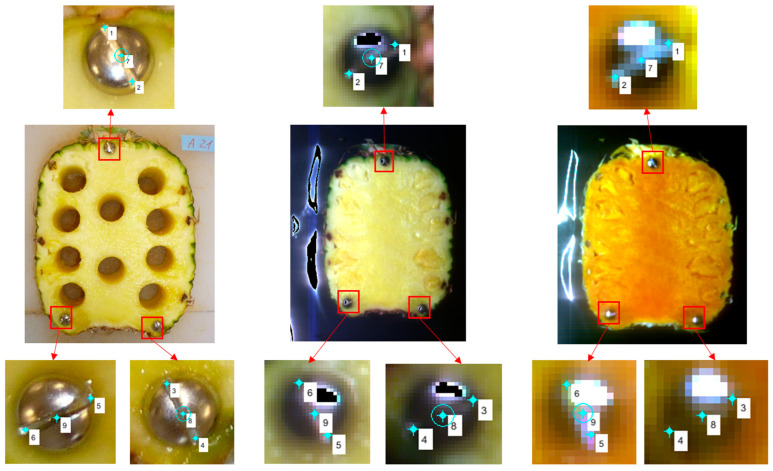
Control points (no. 1 to 6) and check points (no. 7 to 9) for image registration. Left, middle, and right are RGB images of a fruit slice containing the location of samples (holes), 3-channels (460 nm, 550 nm, and 640 nm) image of VIS-SWNIR hyperspectral setup, and 3-channels (1067 nm, 1264 nm, and 1426 nm) image of NIR hyperspectral setup, respectively.

The region of interest was defined by the cylindrical samples extracted from fruit slices after acquiring hyperspectral images. To specify the location of the cylinders on the hyperspectral images, the image taken from the fruit slice after extracting the samples should be registered on the hyperspectral images taken from the same fruit slice (Figure 1). The registration process was performed utilizing a series of control points and transformation functions, which link the corresponding pixels from the distorted image (image taken from the fruit slice after sample extraction) to the reference image (hyperspectral images). Three different types of functions were used for image registration and compared considering the least registration error [22]:

(1) Non-reflective similarity: This transformation is used when shapes in the distorted image are unchanged, but the image is distorted by some combination of translation, rotation, and scaling. Straight lines remain straight, and parallel lines are still parallel.
(2) Affine: It is applied when shapes in the distorted image exhibit shearing. Straight lines remain straight, and parallel lines remain parallel, but rectangles become parallelograms.
(3) Projective: It is used when the scene appears tilted. Straight lines remain straight, but parallel lines converge toward a vanishing point.

Three binding head screws were placed on the fruit slices before taking hyperspectral images in order to extract the control points correctly. The slit at the head of the screws was used as a guideline to extract the control points. A total of 9 points were extracted from each screw in the fruit slice. The location of 6 points was at the beginning and end of the slit at the screws’ head, which were used as control points for registration operations. The other 3 points were selected in the middle of the slit at the screws’ head, which were used as checking points to assess the accuracy of the registration process (Figure 1). The root mean square error (RMSE) and mean absolute percentage error (MAPE) metrics were applied to evaluate the performance of registration functions.

(1) $$RMSE=\sqrt{\frac{\sum_{i=1}^{N}{\left({\hat{y}}_{i}-y_{i}\right)}^{2}}{N}}$$

(2) $$MAPE=\frac{\sum_{i=1}^{N}\left|\frac{{\hat{y}}_{i}-y_{i}}{y_{i}}\right|}{N}\times 100$$

where, $y_{i}$, ${\hat{y}}_{i}$, and N are target (actual) value, output value, and total number of samples, respectively.

**Figure 2:**
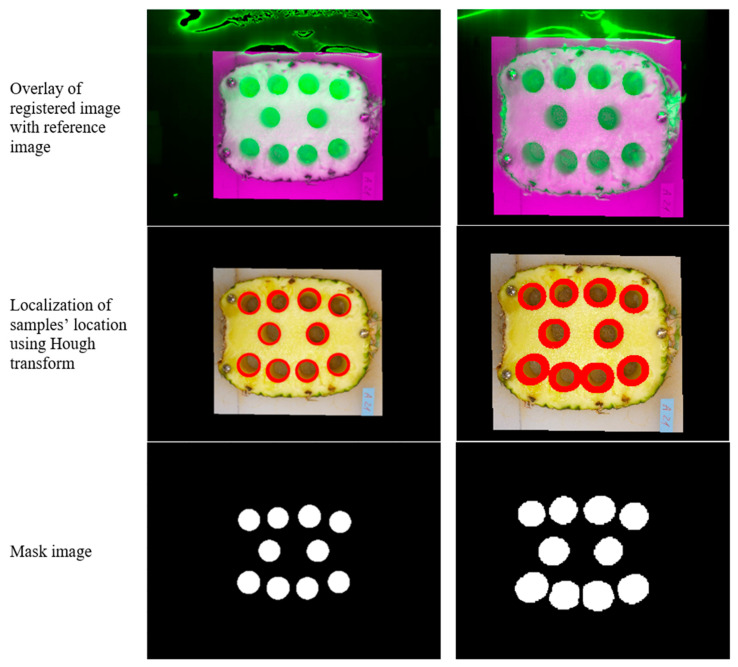
Image registration by affine transformation function and generating mask image capturing the VIS-SWNIR hypercube (**left column**) and the NIR hypercube (**right column**).

Finally, the best registration function was selected considering the lowest value of error metrics for the 60 fruit slices examined, resulting in low registration errors using the affine transformation function (Figure 2). Subsequently, the registered images were processed using the Hough transform method to extract the samples’ location on the slices, which are circular [23]. Since the location of the samples extracted from each fruit slice was determined in the mask image, the mean spectra of the pixels in each segmented sample was used as the spectral signature related to that sample for subsequent analysis. Image registration and mask generation were performed in MATLAB programming environment ver. R2015b (The MathWorks, Inc., Natick, MA, USA). Implementation of the mask image on hypercubes and the extraction of samples mean spectra were performed in ENVI (ver. 4.8, L3HARRIS, Melbourne, FL, USA). 

### 2.4. Spectra Pre-Processing

To isolate the acquired hyperspectral images from the thermal noise of the sensor, the light intensity was corrected using the pure white and black spectra. The black cap of the hyperspectral system lens was placed on it, and the resulting spectrum was saved as the black reference spectrum (*B_H_*). A standard white Teflon sheet with a reflectance of 99% was also used to obtain the white reference spectrum (*W_H_*). Finally, the acquired hyperspectral images (*I_H_*) were corrected (*R_H_*) using the following formula:

(3) $$R_{H}=\frac{I_{H}-B_{H}}{W_{H}-B_{H}}$$

Pixel binning was performed considering all the pixels located in the regions of the fruit slice from which the samples were extracted. Therefore, the mean spectrum of the pixels located in the region corresponding to each sample was taken as the spectral signature of that sample. Also, smoothing based on the Savitzky–Golay algorithm was used to remove random noise from the corrected hyperspectral images. Spectral inspection showed that the spectral points located below 420 nm and above 1600 nm still appeared noisy due to the low photon sensitivity of the camera sensor at both edges. Therefore, the spectral data corresponding to those regions were removed. Since the setup used in this study consists of two hyperspectral imaging units, the spectra acquired from them (the ranges 420–1000 nm of the VIS-SWNIR spectrum and 1000–1600 nm of the NIR spectrum) were integrated into one spectrum in the range 420–1600 nm for subsequent analysis. Pre-processing of hyperspectral images included light intensity correction and further increasing the signal-to-noise ratio performed in MATLAB (ver. R2015b).

### 2.5. Wavelength Selection Process

In this research, lists of commercially available optical equipment for the development of multispectral systems were prepared from the internet (Google search engine, www.google.com, accessed on 8 July 2023) utilizing the keywords “bandpass optical filter”, “LED”, “laser”, and “wavelength” in the range of 420–1600 nm with an interval of 5 nm. One list included bandpass optical filters for filter-based multispectral imaging, and the other list included light sources (LEDs and lasers) for light-based multispectral imaging. Then, for all the acquired spectra of the samples, only the spectral data in the wavelengths that were commercially available were kept. Subsequently, the wavelength selection was performed on the remaining spectral data. In this research, two approaches were considered for selecting optimal wavelengths. The hypothesis used for these approaches is that a good wavelength subset is one that contains wavelengths highly correlated with fruit chemical composition, yet uncorrelated with each other. The wavelength selection process was completed in WEKA (ver. 3.8.6, Waikato Environment for Knowledge Analysis) data mining software [24].

#### 2.5.1. Approach 1: Ranking and Uncorrelatedness

In this approach, the existing wavelengths in the spectrum were ranked according to the highest degree of correlation with the changes in the chemical composition of the fruit by the ReliefF method [25], in descending order. Since highly correlated wavelengths contain identical spectral information, the existence of one of them that has obtained a better rank in correlation with changes in the chemical composition of fruit is sufficient. Therefore, the correlation between the first 20 wavelengths, which had the highest rank according to the ReliefF method, was calculated with each other. Next, the correlation of the first wavelength with the second to twentieth wavelengths was calculated and the wavelengths that had a high correlation with the first wavelength (R > 0.8) were removed. Then, the correlation between the second wavelength and the remaining wavelengths was calculated, and the wavelengths that a had high correlation with the second wavelength were removed. This process was repeated for the remaining wavelengths, and up to the first six ones were introduced as the best wavelengths.

#### 2.5.2. Approach 2: Subset Selection

In this approach, a subset of the wavelengths in the spectrum is extracted using the CfsSubset method [26], which in combination with each other provides the highest correlation with changes in the fruit’s chemical composition. The BestFirst algorithm was used to search the space of wavelength subsets by greedy hillclimbing augmented with a backtracking facility. The value of a subset of features is evaluated, taking into account the individual predictive ability of each feature along with the degree of redundancy between them. A subset of features that are initially highly correlated with the dependent variable (here, fruit chemical composition) and secondly have low intercorrelation with each other is selected as the most appropriate subset. If the number of wavelengths in the subset selected by CfsSubset is more than six, other subsets are generated from it in such a way that all cases where subsets have six members are considered. Then, the correlation of each of the six-member subsets with the changes in the fruit’s chemical composition is calculated. Finally, the subset with the highest correlation is selected as the subset containing the best wavelengths.

### 2.6. Calibration Models

Multilayer perceptron artificial neural networks (MLPs) with four neurons in the hidden layer were used as calibration models to predict the chemical composition of fresh-cut pineapple based on the intensity of reflected light at selected wavelengths as the models’ input and values of reference measurements of chemical composition as their output. The activation function in the hidden layer was chosen as the tangent sigmoid function. The Levenberg–Marquardt algorithm was used to train the networks iteratively. The learning rate, maximum validation failures, mean square error stopping threshold, and the number of epochs of the network were adjusted to 0.3, 15, 0.0001, and 500, respectively. The ‘trial and error’ approach was used to find the best selected wavelength set, considering 20 replications for models’ calibration for prediction of each chemical composition. Since none of the reference measurement data for each origin of pineapple fruits were more spread over the entire range of reference measurements for all origins (Section 3.1), it was not possible to put aside the data from one origin for validation of the models. Instead, before training of the models, two pineapples of 10 fruits per origin were randomly selected in order to use them for validation of the MLP calibration models and to generate distribution maps of chemical composition. Hence, 20% of the total number of samples was included in the internal validation set (2 [fruit/origin] × 10 [sample/fruit] × 6 [origin] = 120). For the rest of fruit, the data from all samples, regardless of their origin, were first randomly shuffled and then it was divided into two parts for calibration (390 samples, 65% of the data) and cross-validation (90 samples, 15% of the data) using the random sample partitioning method. The correlation coefficient (R), RMSE, and MAPE of the calibration and cross-validation stages were then used as a measure of performance to compare models trained with different selected wavelengths set. The models were chosen as the optimal one, which demonstrated the highest R and the lowest RMSE and MAPE in the calibration and cross-validation stages. Finally, the networks with an optimum selected wavelength set were validated on a set of unseen data to confirm their accuracy. Preparing data and creating predictive models were performed in MATLAB ver. R2015b.

### 2.7. Statistical Analysis

Descriptive statistics and significant differences in the chemical composition of pineapples from different origins were obtained using analysis of variance (ANOVA). The mean difference between the chemical composition and all origins was determined by Tukey’s test (*p* < 0.05) using the SAS software (vers. 9.4, SAS Institute, Cary, NC, USA).

## 3. Results and Discussion

### 3.1. Statistical Analysis of Chemical Data

The physical properties of the fruit show no significant difference among the geometric mean diameter of the samples in different origins (*p* < 0.05). Since the height of the samples of Origin 4 and 5 was somewhat larger than other origins, the fruit of Origin 4 and 5 were significantly heavier than other origins (*p* < 0.05). Therefore, in general, the samples in Origins 1 to 3 and Origin 6 were of the same size and weight category (Table 1).

The MC values of pineapples ranged from 85.14 to 88.54% for Origin 1 until Origin 6. Origin 4 (88.54%) recorded the highest MC values of pineapples, followed by Origin 2 (86.70%) and Origin 1 (86.51%), respectively. It was observed that the MC values of pineapples appeared different (*p* < 0.05) for all origins. Similar findings of MC were obtained by Padrón-Mederos et al. [27], ranging between 84.0 and 88.6%. In contrast, Origin 3 (13.76%) had the highest SSC values, with the lowest values recorded by Origin 4 (10.52%). It was also revealed that the SSC values of pineapples were different (*p* < 0.05) considering all origins (Table 1). The SSC values increased in pineapples which could be elucidated by starch conversion to sugar during storage [28]. 

The TA of the pineapples ranged between 0.61 and 0.96% citric acid (Table 1). In addition, the TA values of pineapples differed (*p* < 0.05) for all origins. Origin 4 (0.98% citric acid) recorded the highest TA values of pineapples, followed by Origin 1 (0.96% citric acid) and Origin 2 (0.95% citric acid), respectively. The changes in TA could be attributed to the organic acid formation and degradation reaction during the ripening process of pineapples [29]. On the other hand, the CC values of pineapples ranged from 0.21 to 0.42 mg/100 DM. The lowest CC values were obtained in Origin 6 whereas the highest CC values were found in Origin 2, respectively. The CC values of pineapples had significant differences (*p* < 0.05) for all origins. It can be denoted that the carotenoids’ synthesis determined the progression of the orange or yellow color of the pineapple flesh [30].

### 3.2. Image Registration

Overall, the registration error was low for all transformation functions (MAPE < 2.25%, Table 2). In the two registration modes with VIS-SWNIR images and NIR images, affine transformation showed the best performance and projective transformation showed the worst performance. These results indicate that, compared to the hyperspectral images, the images taken from the fruit slice after extracting the samples suffered from shearing and scaling because the camera is not entirely perpendicular to the surface of the slices and, also, it is closer to the surface of the slices. Therefore, the affine transformation function was used to perform registration operations on hyperspectral images of all fruit slices. Another advantage of the affine function is its ease of use in addition to its higher accuracy. Three control point pairs are required to perform image registration operations using this transformation function. The need for fewer control points increases the speed of image registration operations.

### 3.3. Wavelength Selection

**Figure 3:**
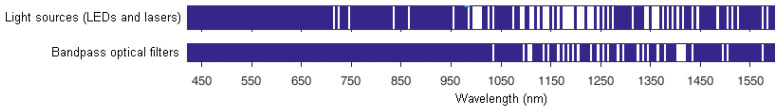
An overview of commercially available optical resources in the range 420–1600 nm (wavelength interval: 5 nm). The available wavelengths are shown in blue.

The spectral data were filtered based on the wavelengths at which light sources were commercially available (Figure 3) and then the optimum wavelengths were identified by “ranking and uncorrelatedness” and “subset selection” approaches (Table 3). The yellow color of pineapple flesh is caused by carotenes and xanthophylls, both groups of which are located in the non-polar parts of the chromoplasts. Carotenes are the more dominant of the two pigment groups [31]. Carotenes and xanthophylls display a strong absorption across the wavelength range from 420 to 515 nm [32]. An increase in the amount of crystallized carotenoids can be found in the vacuoles of fruit and vegetables [33]. However, in neutral (pH 7 and 8) and constant acidity conditions carotenoids are reduced [34]. Maybe more important, the SSC contains the carotenoids to a certain percentage. Overall, results obtained in the current research appear in line with previous work. Based on the destructive reference measurements, R between changes in CC with changes in MC, SSC, and TA was −0.56, 0.52, and −0.14, respectively (*p*-value < 0.01). This shows linearly a decrease and an increase in CC, respectively, when MC and SSC increase. A weak linear relationship between changes in TA and CC indicates the low variation in acidity of samples (coefficient of variation = 0.30). Similar results were observed between spectral data at selected wavelengths and changes in chemical composition of fruit flesh (Table 3). R values between light reflectance data at 485, 490, 495, 500, 505, 510, and 515 nm with values of chemical composition confirm a relatively strong negative, positive, and negative linear relationship between changes in MC, SSC, and TA with light absorption, respectively.

The light absorption at around 960 nm is generally correlated with the second overtone of the OH stretching band of water molecule. Furthermore, spectral reflectance at 1215 nm and 1240 nm is associated with the second overtone of the stretching vibration of sugars and the citric acid CH2 bands. This result is in agreement with the findings of Subedi et al. [35] in determination of sensitive wavebands for assessment of TA in fruit. Carotenoids decrease the membrane fluidity and increase the order of alkyl chains [36]. The 1425 nm vibration is associated with the CH stretching of molecules containing alkyl and aromatic groups and the first overtone of vibration of the OH bond in the water molecule [37].

### 3.4. MLP Prediction Models

The chemical composition of fresh-cut pineapple samples was predicted by MLP models using light reflectance at selected wavelengths (Table 4). The results showed that the R and error metrics were very close between the calibration and cross-validation stages, indicating MLP models are performing well, i.e., good precision, and are not overfitting. The R for calibration and cross-validation indicated a moderate correlation between the predicted values and the reference values for MC, SSC, and CC. Except for CC, the error metrics indicated a low difference in predicted values with the reference ones, especially for MC. The higher values of the error indicate that MLP models are not very reliable for the prediction of CC using selected wavelengths.

Overall, the results showed that for all the studied chemical compositions, the MLP models generated by the selected wavelengths using “ranking and uncorrelatedness” and “subset selection” approaches yielded similar performance both in calibration and cross-validation stages. Since the equipment in the visible wavelength range is more affordable than that in the NIR region, the combination of 495 nm and 500 nm wavelengths may be proposed for predicting MC by both filter-based and light-based multispectral imaging methods. However, the calibration would be partly based on correlation of MC and compounds absorbing in the visible range. Because of higher R and lower RMSE and MAPE values in calibration and cross-validation stages, rationalized by the actual absorption coefficients of OH bonds, 495 nm wavelength and combination of 505 nm and 1425 nm wavelengths are recommended for implementing on filter-based and light-based multispectral imaging systems in order to predict SSC and CC in fresh-cut pineapple, respectively. The best prediction results for TA are obtained by MLP models in the calibration and cross-validation stages when a combination of 500, 505, and 1215 nm wavelengths is used for the filter-based method and a combination of 500, 505, and 1240 nm wavelengths is used for the light-based method. In the case of TA, the relevant wavelengths responsible for the acidity may coincide with other molecules with functional groups OH and CO providing less pronounced calibration. Similar low correlation results for TA were found in many fruits, e.g., tomato [38]. In previous work, SSC was predicted by means of the full spectrum, e.g., providing R = 0.72, while MC reached enhanced correlation coefficients [11,39]. However, the present approach of reduction from full spectrum to the commercially available wavelengths can better support the development of a commercial application.

**Figure 4:**
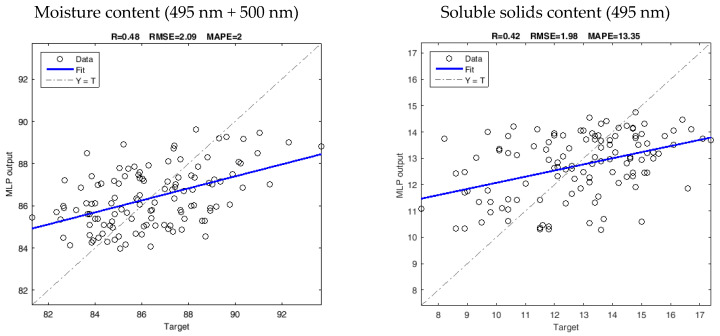

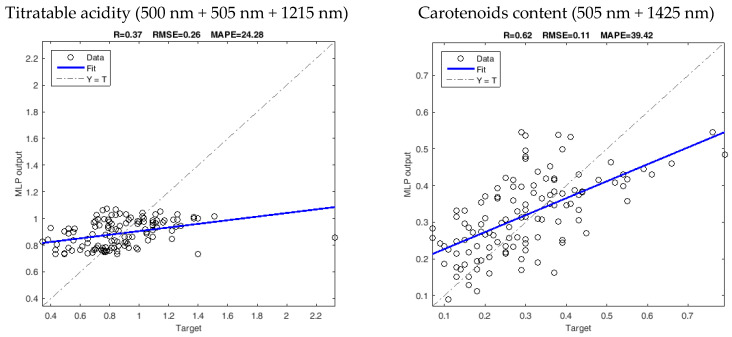
Validation results of MLP models in predicting chemical composition of fresh-cut pineapple based on the superior selected wavelengths.

The MLP models calibrated by superior selected wavelengths were tested using the validation dataset (Figure 4). The results indicated that the models were able to predict the MC (%) and SSC (%) with good accuracy. TA (%) showed a degree of accuracy similar to the cross-validation stage, with values of 0.37 for R, 0.26 for RMSE, and 24.28–24.80% for the MAPE in the validation stage. For CC (mg/100g DM), the error metrics in the validation stage were better than those in the calibration and cross-validation stages, 0.11 for RMSE, and 39.42% for MAPE. These results demonstrate the potential of the models developed in this study to non-invasively predict the parameters of interest, especially for MC and SSC.

### 3.5. Chemical Composition’s Distribution Maps

**Figure 5:**
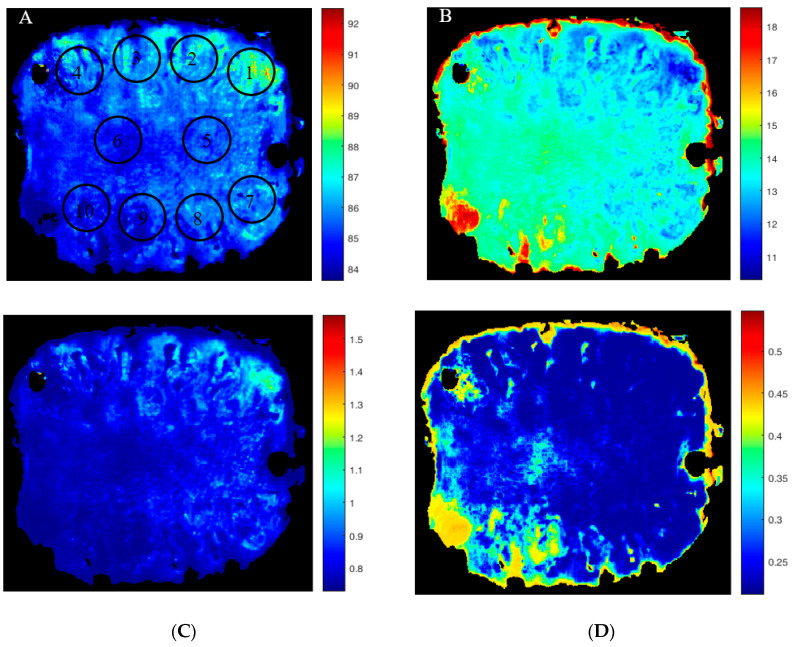
Example of pseudo-color spatial distribution maps of chemical composition for a slice of fresh-cut pineapple, which was separated out for validation of MLP models: (**A**) moisture content in % (495 nm + 500 nm), (**B**) soluble solids content in % (495 nm), (**C**) titratable acidity in g/100 mL (500 nm + 505 nm + 1215 nm), and (**D**) carotenoids content in mg/100g DM (505 nm + 1425 nm). Circles and digits show the regions and their corresponding number, respectively, in which samples were taken for measurement of reference tests.

The main advantage of multispectral imaging compared to other non-destructive optical methods is the ability to create distribution maps of the chemical composition of the product. These maps provide a comprehensive overview of the physiological conditions of the fruit. To do this, by coding in the IDL programming environment of ENVI software, ver. 5.3, hypercubes were converted to two-dimensional (2D) matrices while preserving the spectral information. In each row of the 2D matrices, the spectrum associated with each image pixel is placed. Then, in the MATLAB programming environment, the data related to the matrices containing the reflectance spectrum of the pixels of the image in the superior selected wavelengths (according to the results presented in Section 3.4) were entered into the calibrated MLP models. The output of the models, which is a vector with the same size of image spatial dimension, indicates the predicted chemical composition of the image pixels. To remove the non-useful areas of the image, including the background pixels and screws, a binary mask was created by using the thresholding method in which the pixels of the areas including pineapple slices were valued one and the rest with a value of zero. Next, the mask matrix was converted from the 2D format to a vector of image spatial dimension, the same size as the output vectors of the calibration models. By multiplying the mask matrix in the output vectors of the calibration models, vectors are formed that only contain the predicted values of the desired chemical composition in the areas containing the pineapple slices. Finally, by turning the vectors into matrices with the same size of original images, the distribution maps of the chemical composition for the pineapple slices were obtained (Figure 5). Moisture content showed a gradient from inner to outer parts due to the central stem axis with slightly lower moisture content. More important, the maturity gradient, indicated by the SSC and by the CC, appeared along the stem axis, which is usually seen, due to the difference in ripening time of each fruitlet as determined by the time of flowering in the inflorescence. Such gradient challenges the production of fresh-cut produce. Having a low-cost system available would reduce food waste and enhance consistent product quality within a single fresh-cut package.

For all the fruit considered as the internal validation data set, spatial distribution maps were generated and the mean absolute percentage of the difference of the mean values of predicted chemical compositions of pixels within each of ten regions of interest, where samples were taken for measurement of reference tests, with the actual values of chemical composition at those regions was calculated (Table 5). The mean comparison using Tukey’s test showed no difference among the errors of MLP models in predicting chemical composition spatially throughout the fruit slices (*p* < 0.05). This shows not only the generalizability of the calibration models but also the robustness of their responses in predicting the chemical composition throughout the slices and its feasibility to measure the practically relevant intra-fruit differences. The spatial distribution maps provide the possibility to monitor the surface of fruit slices completely. A histogram can be generated from these maps to provide a visual representation of the pixel distribution within the fruit slice, which can aid in the interpretation of the prediction image. The shape and offset of the histograms (quantitatively as mean, skewness, and kurtosis) can be used as criteria to identify whether a slice of pineapple is acceptable in terms of quality or not. Accordingly, such technology can be implemented on the cutting machines to ensure that only fruit parts with the desired quality are packaged together.

## 4. Conclusions

Different levels of quality can be seen in pineapples by means of hyperspectral imaging, which is usually seen along the stem axis, due to the difference in ripening time of each fruitlet as determined by the flowers in the inflorescence. This can make it difficult to achieve consistent quality within a single fresh-cut package. To address this issue, the postharvest industry requests non-contact imaging systems to analyze the quality of pineapple slices spatially. This will help identify any differences in quality that may exist along the stem axis, thereby helping to ensure uniform quality across the entire package and avoid food waste due to earlier decayed fruit pieces in otherwise marketable packaged fruit pieces. 

In this research, a practical approach was presented which can be used to set up filter-based or light-based multispectral imaging systems to create spatial distribution maps of chemical compositions over the surface of pineapple slices. This was achieved initially by preparing a list of wavelengths in the range of 420–1600 nm in which optical equipment is commercially available. Then, dimensionality reduction methods show a high sensitivity to changes in chemical compositions. Accordingly, it has been determined that [495, 500, 505, 1215, and 1425 nm] and [495, 500, 505, 1240, and 1425 nm] are the most suitable set of wavelengths for setting up filter-based and light-based multispectral imaging systems, respectively. The rate of linearity for the results of calibrated MLP models was for CC (R = 0.63), MC (R = 0.56), and SSC (R = 0.52). Furthermore, the calibration models were able to predict MC (MAPE = 1.92%) and SSC (MAPE = 14.72%) with a low error. However, due to the high error in prediction, the calibration models were not of sufficient confidence to predict CC (MAPE = 43.99%). Compared to the MLP models created by the first four principal components of the whole spectra (420–1600 nm) on the same dataset, the performance of the calibration models created by the selected wavelengths is acceptable, since similar results was yielded in terms of linearity and the error rate of prediction for MC and SSC. Statistical analysis revealed that there is no difference between the error rates of MLP models spatially across the surface of the fruit slices considering ten spatially distributed references per fruit slice. This is an indication of the reliability of calibration models in mapping the spatial distribution of chemical compositions in fresh-cut pineapple. 

The results show that the variability of chemical composition can be analyzed by means of the feasible solution proposed. Further research and in-depth analysis will be needed to develop more precise and robust models, especially for prediction of CC.

## Figures and Tables

**Table 1:** Descriptive statistics of chemical composition of pineapples (N = 60) from different origins.

Origin No.	Mass (g)	Height (cm)	Arithmetic Mean Diameter (cm)	Moisture Content (MC, in %)	Soluble Solids Content (SSC, in %)	Titratable Acidity (TA, in %)	Carotenoids Content (CC, in mg/100 g DM)
Origin 1	1146 ^b^ ± 337	14.30 ^bc^ ± 2.57	10.02 ^a^ ± 0.64	86.51 ^b^ ± 1.34	13.02 ^a^ ± 1.19	0.96 ^a^ ± 0.07	0.34 ^ab^ ± 0.12
Origin 2	1053.2 ^b^ ± 88.6	12.80 ^c^ ± 0.95	13.37 ^a^ ± 9.95	86.70 ^ab^ ± 2.24	12.42 ^ab^ ± 2.19	0.95 ^a^ ± 0.21	0.42 ^a^ ± 0.05
Origin 3	1165.5 ^b^ ± 84.7	13.75 ^bc^ ± 0.49	10.33 ^a^ ± 0.55	85.14 ^b^ ± 1.81	13.76 ^a^ ± 1.17	0.92 ^a^ ± 0.15	0.35 ^a^ ± 0.12
Origin 4	1604.6 ^a^ ± 133.8	16.60 ^a^ ± 0.99	11.03 ^a^ ± 0.63	88.54 ^a^ ± 1.28	10.52 ^b^ ± 1.63	0.98 ^a^ ± 0.16	0.23 ^bc^ ± 0.10
Origin 5	1521 ^a^ ± 377	15.55 ^ab^ ± 2.09	10.83 ^a^ ± 0.90	86.13 ^b^ ± 0.58	12.92 ^a^ ± 0.53	0.61 ^b^ ± 0.14	0.35 ^ab^ ± 0.09
Origin 6	1358 ^ab^ ± 320	14.00 ^bc^ ± 1.87	11.07 ^a^ ± 0.73	86.35 ^b^ ± 1.10	13.18 ^a^ ± 0.91	0.93 ^a^ ± 0.17	0.21 ^c^ ± 0.11
All origins	1308 ± 316.2	14.50 ± 2.01	11.11 ± 4.05	86.56 ± 1.75	12.64 ± 1.74	0.89 ± 0.19	0.32 ± 0.10

Data represents the mean ± standard deviation. Different letters within the same column indicate statistical difference by the Tukey’s test (*p* < 0.05).

**Table 2:** The error metrics in control and checking points for evaluating three transformation functions applied for image registration of pineapple slices.

Transformation Function	VIS-SWNIR				NIR			
	RMSE_Reg_ (Pixel)	MAPE_Reg_ (%)	RMSE_Ch_ (Pixel)	MAPE_Ch_ (%)	RMSE_Reg_ (Pixel)	MAPE_Reg_ (%)	RMSE_Ch_ (Pixel)	MAPE_Ch_ (%)
Non-reflective similarity	2.85 ± 1.15	0.52 ± 0.17	3.09 ± 1.81	0.56 ± 0.26	2.21 ± 0.65	0.84 ± 0.27	2.25 ± 1.02	0.82 ± 0.25
Affine	1.48 ± 1.29	0.25 ± 0.14	1.81 ± 1.84	0.28 ± 0.16	1.17 ± 0.45	0.45 ± 0.16	1.29 ± 1.02	0.49 ± 0.32
Projective	2.40 ± 3.94	0.43 ± 0.69	2.82 ± 3.19	0.55 ± 0.65	1.25 ± 0.83	0.48 ± 0.32	2.45 ± 2.83	1.02 ± 1.22

Data represents the mean ± standard deviation of 10 fruit slices. RMSE_Reg_, MAPE_Reg_, RMSE_Ch_, and MAPE_Ch_ stand for root mean square error of registration (control) points, mean absolute percentage error of registration points, root mean square error of checking points, and mean absolute percentage error of checking points, respectively.

**Table 3:** Selected wavelength based on “ranking and uncorrelatedness” and “subset selection” approaches for the dataset contacting the commercially available wavelengths of bandpath optical filters and lights sources.

Chemical Composition	Bandpath Optical Filters		Light Sources	
	Ranking and Uncorrelatedness	Subset Selection	Ranking and Uncorrelatedness	Subset Selection
Moisture content (MC, in %)	490 nm (0.54) *, 1565 nm (0.07)	495 nm (0.54), 500 nm (0.54)	490 nm (0.54), 1565 nm (0.07)	495 nm (0.54), 500 nm (0.54)
Soluble solids content (SSC, in %)	485 nm (−0.49)	495 nm (−0.49)	485 nm (−0.49)	495 nm (−0.49)
Titratable acidity (TA, in %)	515 nm (0.32), 960 nm (0.19)	500 nm (0.34), 505 nm (0.34), 1215 nm (0.26)	515 nm (0.32), 960 nm (0.19)	500 nm(0.34), 505 nm (0.34), 1240 nm (0.25)
Carotenoids content (CC, in mg/100g DM)	510 nm (−0.53)	505 nm (−0.55), 1425 nm (0.21)	510 nm (−0.53)	505 nm (−0.55), 1425 nm (0.21)

* The digits in parentheses show the correlation of light reflectance at that wavelength with each chemical composition.

**Table 4:** Prediction of chemical composition of fresh-cut pineapple by MLP models using selected wavelengths in the calibration and cross-validation stages.

Statistical Measure	Moisture Content (MC)		Soluble Solids Content (SSC)		Titratable Acidity (TA)			Carotenoids Content (CC)	
	490 nm + 1565 nm	495 nm + 500 nm	485 nm	495 nm	515 nm + 960 nm	500 nm + 505 nm + 1215 nm	500 nm + 505 nm + 1240 nm	510 nm	505 nm + 1425 nm
R_c_	0.59 ± 0.03	0.58 ± 0.03	0.50 ± 0.04	0.50 ± 0.03	0.33 ± 0.03	0.37 ± 0.03	0.37 ± 0.05	0.54 ± 0.02	0.65 ± 0.02
RMSE_c_	2.05 ± 0.07	2.05 ± 0.07	2.09 ± 0.05	2.09 ± 0.06	0.26 ± 0.02	0.26 ± 0.02	0.26 ± 0.02	0.13 ± 0.00	0.12 ± 0.00
MAPE_c_	1.90 ± 0.07	1.89 ± 0.07	14.97 ± 0.52	14.88 ± 0.62	23.02 ± 0.97	22.29 ± 2.09	22.72 ± 1.05	50.43 ± 2.70	44.45 ± 2.58
R_cv_	0.55 ± 0.05	0.56 ± 0.05	0.49 ± 0.07	0.52 ± 0.07	0.37 ± 0.06	0.35 ± 0.06	0.37 ± 0.09	0.53 ± 0.05	0.63 ± 0.04
RMSE_cv_	2.11 ± 0.11	2.10 ± 0.15	2.99 ± 3.95	2.07 ± 0.11	0.24 ± 0.03	0.25 ± 0.03	0.25 ± 0.04	0.13 ± 0.01	0.12 ± 0.01
MAPE_cv_	1.93 ± 0.09	1.92 ± 0.12	14.80 ± 1.04	14.72 ± 1.26	23.05 ± 2.18	23.53 ± 1.95	23.70 ± 2.15	49.61 ± 4.60	43.99 ± 5.08

Data represent the mean ± standard deviation of 20 replicates. R_c_ and R_cv_, RMSE_c_ and RMSE_cv_, and MAPE_c_ and MAPE_cv_ are the correlation coefficient, root mean square error, and mean absolute percentage error for calibration and cross-validation stages, respectively.

**Table 5:** Mean absolute percentage of the difference between mean values of predicted chemical composition of pixels within 10 different regions of the slices with the actual values of chemical composition for 12 fruits in the validation dataset. The location of measuring spots has been shown in Figure 5A.

Chemical Composition	Region No.									
	1	2	3	4	5	6	7	8	9	10
Moisture content (495 nm + 500 nm)	2.39 ^a^ ± 1.15	1.54 ^a^ ± 1.28	2.41 ^a^ ± 1.82	2.52 ^a^ ± 1.51	2.53 ^a^ ± 1.30	1.76 ^a^ ± 1.22	2.17 ^a^ ± 1.23	1.39 ^a^ ± 1.02	1.61 ^a^ ± 1.23	1.67 ^a^ ± 1.48
Soluble solids content (495 nm)	16.25 ^a^ ± 11.10	11.86 ^a^ ± 5.31	11.26 ^a^ ± 6.40	13.52 ^a^ ± 9.53	21.69 ^a^ ± 19.19	18.54 ^a^ ± 15.38	16.86 ^a^ ± 14.48	8.48 ^a^ ± 5.69	7.50 ^a^ ± 5.59	7.48 ^a^ ± 6.93
Titratable acidity (500 nm + 505 nm + 1215 nm)	27.88 ^a^ ± 31.63	20.83 ^a^ ± 21.22	18.70 ^a^ ± 17.16	18.06 ^a^ ± 17.43	26.07 ^a^ ± 20.16	24.05 ^a^ ± 21.79	28.84 ^a^ ± 37.62	32.21 ^a^ ± 40.01	24.64 ^a^ ± 26.71	21.03 ^a^ ± 20.21
Titratable acidity (500 nm + 505 nm + 1240 nm)	26.31 ^a^ ± 32.81	22.10 ^a^ ± 20.58	21.47 ^a^ ± 14.76	18.13 ^a^ ± 16.16	26.83 ^a^ ± 20.68	25.45 ^a^ ± 23.16	28.48 ^a^ ± 42.04	31.90 ^a^ ± 43.28	25.53 ^a^ ± 29.63	21.20 ^a^ ± 20.04
Carotenoids content (505 nm + 1425 nm)	27.53 ^a^ ± 15.40	28.50 ^a^ ± 17.80	19.43 ^a^ ± 17.51	32.31 ^a^ ± 38.66	76.04 ^a^ ± 89.12	70.63 ^a^ ± 70.57	52.15 ^a^ ± 49.40	27.63 ^a^ ± 35.82	27.33 ^a^ ± 20.90	34.12 ^a^ ± 25.52

Data represents the mean ± standard deviation of 12 samples. Same letters within the same row indicate no statistical difference by the Tukey’s test (*p* < 0.05).

## Data Availability

The data used to support the findings of this study can be made available by the corresponding author upon request.
